# Impacts caused by the use of screens during the COVID-19 pandemic in children and adolescents: an integrative review

**DOI:** 10.1590/1984-0462/2024/42/2022181

**Published:** 2023-10-23

**Authors:** Maria Alice Aparecida Resende, Mariana Luiza da Fonseca, Jéssica Tertuliano de Freitas, Elaine Cristina Rodrigues Gesteira, Lisabelle Mariano Rossato

**Affiliations:** a Universidade Federal de São João del-Rei, Campus Centro Oeste, Divinópolis, MG, Brazil.; b Universidade de São Paulo, São Paulo, SP, Brazil.

**Keywords:** Child development, Mobile applications, Exposure time, Risk factors, Coronavirus infections, Desenvolvimento infantil, Aplicativos móveis, Tempo de exposição, Fatores de risco, Infecções por Coronavírus

## Abstract

**Objective:**

To identify the scientific evidence on the impacts caused by the use of screens during the COVID-19 pandemic in children and adolescents, raising reflections for future interventions with this public.

**Data source:**

This is an integrative literature review, conducted in the databases Medical Literature Analysis and Retrieval System Online (MEDLINE), Literatura Latino-Americana e do Caribe em Ciências da Saúde (LILACS), Cumulative Index to Nursing and Allied Health Literature (CINAHL), United States National Library of Medicine (PubMed), Scopus, Web of Science, and Embase, published from March 2020 to January 2022, in Portuguese, English and Spanish.

**Data synthesis:**

The search strategies allowed retrieving 418 articles, of which 218 were duplicates. The analysis of titles and abstracts resulted in the maintenance of 62 studies. Of these, 31 were excluded from the reading of the full text, since they did not clearly present the phenomenon investigated. Thirty-one were eligible, resulting in five categories: eye consequences; increased sedentary behavior and weight; change in eating habits; implications for sleep quality and impacts on mental health.

**Conclusions:**

The excessive use of screens during the pandemic led to numerous consequences for children and adolescents, with a higher incidence of visual damage, sedentary lifestyle, inadequate eating habit and increased weight gain, in addition to impaired sleep quality and mental health. This study provides subsidy for health professionals to carry out continuing education focused on this theme, and elaborate effective interventions for this public in this transition to the post-pandemic period.

## RESUMO

**Objetivo:**

Identificar as evidências científicas sobre os impactos causados pelo uso de telas durante a pandemia da COVID-19 em crianças e adolescentes, almejando reflexões para futuras intervenções com esse público.

**Fontes de dados:**

Trata-se de uma revisão integrativa da literatura, realizada nas bases de dados Medical Literature Analysis and Retrieval System Online (MEDLINE), Literatura Latino-Americana e do Caribe em Ciências da Saúde (LILACS), Cumulative Index to Nursing and Allied Health Literature (CINAHL), United States National Library of Medicine (PubMed), Scopus, Web of Science e Embase, publicados de março de 2020 a janeiro de 2022, em português, inglês e espanhol.

**Síntese dos dados:**

As estratégias de busca possibilitaram recuperar 418 artigos, sendo 218 duplicados. A análise de títulos e resumos resultou na manutenção de 62 estudos. Destes, após a leitura do texto integral foram excluídos 31, uma vez que não apresentavam com clareza o fenômeno investigado. Foram elegíveis 31 estudos, emergindo cinco categorias: consequências oculares; aumento do comportamento sedentário e do peso; alteração dos hábitos alimentares; implicações na qualidade do sono e impactos na saúde mental.

**Conclusões:**

Percebe-se que o uso telas em excesso durante a pandemia trouxe inúmeras consequências para o público infantojuvenil, com maior incidência de acometimentos visuais, sedentarismo, alimentação inadequada e, por consequência, maior ganho de peso, além de prejuízos à qualidade do sono e à saúde mental. Este estudo fornece subsídios para que os profissionais da saúde realizem educação continuada voltada para essa temática e elaborem intervenções efetivas para esse público nesta transição para o período pós-pandêmico.

## INTRODUCTION

Technologies evolve rapidly, and are inserted in the daily lives of children and adolescents through pocket, mobile and portable devices, having consequences for healthy development.^1^ In childhood, heavy use of these devices may increase the risk of cardiovascular diseases and psychological disorders, in addition to favoring exposure to inadequate content. Some authors associate prolonged screen exposure to delays in linguistic domains and fine motor skills.^1^ In adolescence, it significantly influences social and family interactions and mood, with a greater risk of developing depression, self-extermination attempts, low self-esteem, as well as other behavioral problems.^2^

Children's early access to technologies and the inappropriate use of these tools can compromise their social, cognitive, and affective development. In these media, they are exposed to advertisements that rely on persuasive and appealing techniques to reach mainly children, exercising intense control over their behavior.^3^ The trajectory of screen use was analyzed at 24, 36 and 60 months of age in Canadian children, and heavy use showed poorer child development and sub-optimal learning outcomes.^4^ In addition, intense use of screens contributes to weight gain that can trigger obesity, sedentarism, and accelerate or accentuate possible physical and mental disorders.^4,5^

Use of screens, including television sets, video games, and computers, is a practice that works as a source of leisure and entertainment for adolescents, replacing outdoor activities. The impact of this habit results in consumption of unhealthy foods and physical inactivity, increasing the prevalence of overweight and obesity in this age group. In addition, excessive use of these technologies can have negative consequences on vision development, as well as on sleep quality and psychological health.^6^

Such a reality and its problems have been intensified with the advent of COVID-19. In March 2020, the World Health Organization declared the Coronavirus Disease 2019 (COVID-19) pandemic — an acute, potentially serious respiratory infection with high transmissibility^7^ that required necessary global preventive measures, such as social distancing to break the disease's transmission chain. Currently, the impacts of these restrictions on the population's health, communication, interpersonal, family, social and economic relationships can be observed.^4^

Initially, with the interruption of school activities, children and adolescents witnessed abrupt changes in their daily routines, were physically more distant from friends, remained in home confinement, and were more exposed to alarming information about the pandemic, increasing feelings of anxiety, stress and sadness.^4^ There was an absence of school space and decreased outdoor leisure activities, increasing the use of screens and affecting quality of life. This new reality presents short, medium and long-term effects directly linked to the development of comorbidities, in addition to physical, psychological and behavioral complications that may arise.^8^

In January 2021 the vaccination campaign against COVID-19 began in Brazil, in a gradual progress, and in 2022 the vaccine was made available to children.^9^ With the advancement of vaccination and its benefits, there was a decrease in restrictive measures, including the return to school. However, studies show that even after going back to school children have low levels of physical activity and increased screen time and sedentary activities.^5^

This study is justified by the need to investigate the impacts caused by screen use during the pandemic in children and adolescents, grouping information essential for the performance of health professionals given the repercussions of the COVID-19 pandemic. Thus, it aimed to identify scientific evidence of the impacts of screen use during the COVID-19 pandemic in children and adolescents, raising reflections for future interventions with this public.

## METHOD

This is a study that used the integrative review, broad method, which combines experimental and non-experimental research to achieve a more didactic understanding of a phenomenon of interest, presenting the state of science and applicability in health practice.^10^

For the elaboration of this integrative review, the following steps were performed: identification of the problem (clear definition of the review purpose), search of the literature (delimitation of keywords, databases, establishment of inclusion and exclusion criteria for the selection of articles), evaluation and data analysis.

To guide the integrative review, the following question was formulated: What are the impacts caused by the use of screens during the COVID-19 pandemic on children and adolescents?

The following electronic databases were used: Medical Literature Analysis and Retrieval System Online (MEDLINE), Latin American and Caribbean Literature in Health Sciences (LILACS), Cumulative Index to Nursing and Allied Health Literature (CINAHL), National Library of Medicine and National Institutes of Health (PubMed), Scopus, Web of Science, and Embase. The search was conducted by the authors independently in order to ensure the legitimacy of the study, recovering scientific articles published from March 2020, after the beginning of the pandemic and the consequent establishment of prevention measures.

The inclusion criteria of the articles were: original, complete studies, published in English, Portuguese and Spanish, with full texts, available online in the selected databases, addressing the impacts of the use of screens on children and adolescents during the COVID-19 pandemic in the period from March 2020 to January 2022. This is the period between the recognition by the World Health Organization of COVID-19 as a pandemic and the date of the search for articles in the databases.^11^ Articles that did not include the guiding question, review, abstracts and conference proceedings, editorials, experience reports, theoretical essays, single case study and duplicate studies were excluded from the selection.

The search was conducted during the month of January 2022, using combinations between the keywords, which are descriptors in Descriptors in Health Sciences (DeCS), Medical Subject Headings (MeSH) and Embase subject headings (Emtree): Screen Time/Tempo de Tela/Tiempo de Pantalla and Child/Criança/Niño and Adolescent/Adolescente/Adolescente, combined with the term COVID-19 in all three languages.

The studies retrieved by searching the databases were exported to the Rayyan QCRI Program. Subsequently, duplicate studies were removed, and titles and abstracts were read by three reviewers (MAAR, MLF e JTF) using the double-blind method in order to identify potentially eligible studies. Those studies that presented doubts and/or divergences between the reviewers regarding the inclusion or exclusion were solved by a fourth author with expertise in child and adolescent health (ECRG).

For the compilation of results, a table was built with the main characteristics of the studies, such as: title, authors and year of publication, journal, language, country of origin, objective, methodological approach, population, main results, conclusions, and bibliographic references.

There was no need for approval by the Research Ethics Committee because this is a bibliographic study. Ethical aspects and copyright were respected, and the studies referenced.

The articles included in this study were selected, evaluated, and classified hierarchically according to their level of evidence. This practice contributes to the decision-making of health professionals. In this study, the proposal of Melnyk and Fineout-Overholt was adopted, which is shown in Table 1.

**Table 1:** Level of evidence classification.

I	Evidence from systematic review or meta-analysis of all relevant randomized controlled trials or from clinical guidelines based on systematic reviews of randomized controlled trials.
II	Evidence derived from at least one well-designed randomized controlled clinical trial.
III	Evidence obtained from well-designed clinical trials without randomization.
IV	Evidence from well-designed cohort and case-control studies.
V	Evidence from a systematic review of descriptive and qualitative studies.
VI	Evidence derived from a single descriptive or qualitative study.
VII	Evidence from opinion of authorities and/or report of expert committees.

## RESULTS

**Figure 1:**
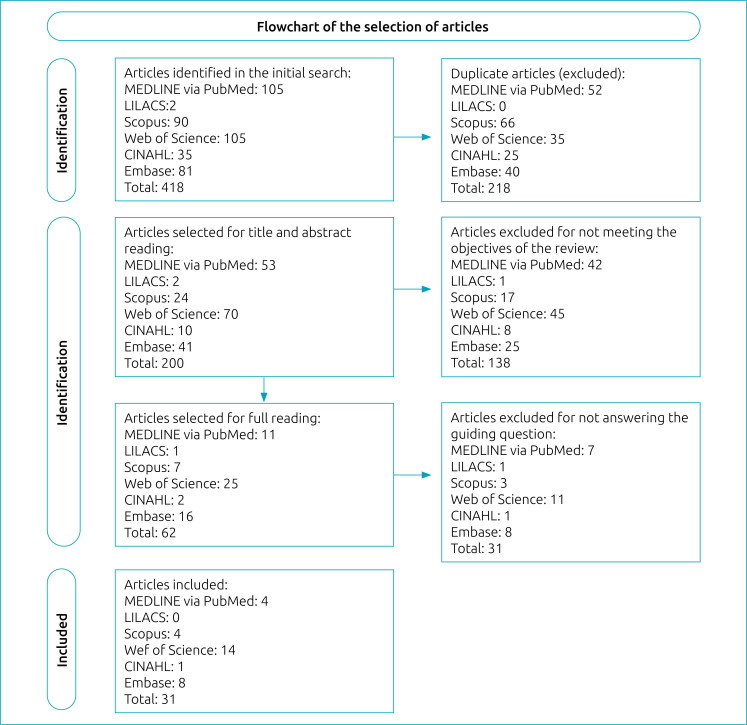
Flowchart of article selection.

The search strategies allowed retrieving 418 articles, of which 218 were duplicates. The analysis of titles and abstracts resulted in 62 studies being included. Of these, 31 were excluded from reading of the full text, since they did not clearly present the phenomenon investigated, and 31 were included in the review, as shown in the flowchart (Figure 1).

Of the 31 papers analyzed, all were quantitative articles, except one. As for year of publication, the highest incidence was 2021 (27 articles), 2020 (2 articles) and 2022 (2 articles). Seven were developed in China, four in Canada, two in Italy, India, France and Turkey, and one in the Netherlands, Spain, Japan, Brazil, Saudi Arabia, Iran, the United States of America (USA), Hungary, Jordan, Indonesia, Poland and Egypt. All studies were published in English. The table shows the studies’ main characteristics (Table 2).^2,5,6,12-39^

**Table 2:** Characterization of articles according to author, year of publication, type of study, methodological approach, and level of evidence.

Reference	Country	Type of study	Level of evidence
McArthur et al.^12^	Canada	Longitudinal cohort (n=846)	IV
Ten Velde et al.^5^	Netherlands	Exploratory cohort (n=233)	IV
Liu et al.^13^	China	Prospective cohort (n=3831)	IV
Choi et al.^14^	China	Exploratory cohort (n=171)	IV
Zhang et al.^15^	China	Prospective cohort (n=1793)	IV
Adachi et al.^16^	Japan	Prospective cohort (n=4875)	IV
Li et al.^17^	Canada	Longitudinal cohort (n=2026)	IV
Alghadir et al.^18^	Saudi Arabia	Prospective cohort (n=214)	IV
Saxena et al.^19^	India	Prospective cohort (n=1237)	IV
Cardy et al.^20^	Canada	Prospective cohort (n=414)	IV
Xiao et al.^2^	China	Prospective cross-sectional (n=1680)	IV
Pietrobelli et al.^21^	Italy	Observational longitudinal (n=32)	IV
Kołota and Głąbska^22^	Poland	Longitudinal (n=1334)	IV
Hartley et al.^23^	France	Longitudinal (n=1777)	IV
Alvarez-Peregrina et al.^24^	Spain	Cross-sectional (n=1600)	V
Helito et al.^25^	Brazil	Cross-sectional (n=387)	V
Shuai et al.^26^	China	Cross-sectional (n=192)	V
Oliva et al.^27^	Italy	Cross-sectional prospective (n=9974)	V
Surekha et al.^28^	India	Cross-sectional (n=2000)	V
Hadianfard et al.^6^	Islamic Republic of Iran	Cross-sectional (n=510)	V
Tandon et al.^29^	United States	Cross-sectional l (n=1000)	V
Peddie et al.^30^	Canada	Cross-sectional (n=146)	V
Guo et al.^31^	China	Descriptive cross-sectional (n=10,416)	V
Berki and Pikó^32^	Hungary	Cross-sectional (n=705)	V
Wang et al.^33^	China	Prospective cross-sectional (n=123,535)	V
Chambonniere et al.^34^	France	Cross-sectional (n=6491)	V
Hourani et al.^35^	Jordan	Cross-sectional (n=477)	V
Andriyani et al.^36^	Indonesia	Qualitative (n=20)	V
Ceylan et al.^37^	Türkiye	Descriptive cross-sectional (n=384)	V
Yakşi et al.^38^	Türkiye	Cross-sectional (n=506)	V
Hashem et al.^39^	Egypt	Cross-sectional (n=765)	V

All the papers examined addressed the theme of the impacts of screen use during the COVID-19 pandemic on children and adolescents. Five categories emerged from these findings: eye consequences; increased sedentary behavior and weight; altered eating habits; implications for sleep quality and impacts on mental health.

### Category one — eye consequences

The studies analyzed revealed the association between high use of screens and higher risks of myopia, as well as other eye problems in children and adolescents during the COVID-19 pandemic.^13-15,19,24,30,33^

Chinese researchers carried out a large-scale survey, finding that the use of screens in this period increased due to school closures, as well as myopia was more prevalent among respondents who were in high school and fundamentally, screen time was associated with greater risks of symptomatic myopia progression and the study population used more computers and smartphones.^13^ The researchers also claim, that the use of computers and smartphones has a higher probability of facilitating the progression of myopia compared to the use of television for remote education.^13^

Similar results were found in other studies regarding the progression and incidence of myopia associated with restrictive measures to contain the spread of Covid-19, in addition to axial stretching.^14,15^ An association was also found between excessive exposure to electronics eyestrain, blurred vision or dry eyes.^31^

### Category two — increased sedentary behavior and weight

The studies revealed greater use of screens during the period of restrictive measures to control COVID-19, providing increased sedentary activities, decreased physical exercise and less time outdoors, compared to the year before the pandemic.^5,6,15,18,19,21,24,28,34,37^ Furthermore, there was an increase in children's weight and body mass index (BMI) due to longer time watching television and using computers.^18,21,28,37^

With the restrictions imposed, the increased use of screens was inevitable, since they became educational tools and a form of leisure, especially for those with restricted access to external areas.^31,34,36^ Electronic devices, whether televisions, computers, laptops or tablets, when located in bedrooms, present a greater risk of being used for long periods.^18^

The alarming fact is that, even after relaxation of the restriction measures, children still reported performing fewer physical activities than in the previous year, showing that the reopening of schools did not fully restore these activities. This indicates that there may be a lasting impact related to screen use time in that such behavior has been normalized.^5^ Therefore, ending the COVID-19 restriction measures alone will not by itself promote new habits in children and adolescents.^5^

### Category three — altered eating habits

Another issue analyzed in the studies was the diet associated with the use of electronics, where a common habit of eating while watching TV was found.^35^ This is related to higher fat intake and consumption of advertised products, such as sugary cereals, sweets, drinks and snacks.^28^ Such behavior was more observed as of the start of distance education during the pandemic, compared to the previous year.^22^ The use of screens can indirectly increase food consumption and the intake of unhealthy foods, reduce physical activity and energy expenditure, increasing the risk of obesity.^6^

Investigating the relationship between screen time and changes in the diet of children and adolescents showed that increased use of mobile phones, television, laptop and video games was associated with increased appetite, increased consumption of sweets and unhealthy foods, snacks and frequent snacks between meals, as well as with children who were indifferent to eating fruits and vegetables.^39^ Scientific evidence suggests that the change in diet and lifestyle caused by the pandemic has led to excessive body weight gain in children and adolescents, and this can have serious health consequences.^21^

### Category four — implications for sleep quality

The excessive use of electronics, according to the studies analyzed, has consequences on hygiene and sleep quality, especially when these devices are used at night.^23,25^ This can be explained due to changes in the biological clock, which plays an important role in the good performance of restorative sleep.^23^ Night exposure to intense light can stimulate the wakefulness system, making individuals more alert and causing them to sleep later.^23^

Poor sleep quality may be related to the exacerbated use of screens, changes in routines and schedules, little exposure to light during the day and a lot of exposure to light at night, changes that may be related to the restriction measures imposed during the pandemic,^23^ bringing physical and psychological consequences to children and adolescentes.^12^

### Category five — impacts on mental health

The changes caused by social isolation had a great impact on the mental health of the pediatric and adolescent population.^12,20,27,29,38^ This new reality implies less time for performing outdoor activities and lack of socialization with peers, consequently fostering the use of electronic devices both for education and as a form of leisure.^17,27^

Children who use screens for long periods have higher levels of depressive and anxiety symptoms.^12,17,26-28,32,38^ In contrast, although education implies spending several hours in front of a screen, learning can act as a form of protection, since it allows the reduction of stressors caused by confinement.^27^

The use of smartphones at younger ages may be a risk factor for mental health in the context of the COVID-19 pandemic.^16^ Therefore, it is necessary to adopt measures aimed at minimizing damage, using other devices in a moderate way and with limited time.

Studies show that more time on TV or digital media causes high levels of inattention and hyperactivity, and the excessive use of video games provoke irritability, inattention and hyperactivity.^26,27^ In addition, longer screen time is associated with more conflicts with parents, since this habit can lead to a negative mood in adolescents.^2^

## DISCUSSION

With globalization, the use of technologies has become commonplace, and this reality has both positive and negative aspects; in the pandemic period experienced in recent years, this scenario has become more evident.^6^ Although in developed countries access to electronic devices is easier compared to developing countries, among the studies analyzed, the largest amount of research was conducted in emerging countries (n=17), showing that the impacts of screen use have been a global concern, also confirmed by the diversity of countries developing research on the subject.

The more frequent access of children and adolescents to the web is not a recent concern, but has intensified in several countries including Brazil, since the problematic use of these devices can cause mental, visual and auditory disorders.^13,16,25^ In 2019, Brazilians were ranked third in relation to daily hours online,^40^ Brazilian teenagers in particular ranked second in internet connection time outside of school hours.^41^ However, only one of the studies analyzed here was developed in Brazil, showing the need to develop more research on the subject, especially after the pandemic.

As addressed in category one, although myopia is considered a benign alteration, its prevalence has become increasingly common. This pathology can lead to severe visual complications, including macular degeneration, posterior staphyloma, cataract, glaucoma, and retinal detachment, becoming a public health concern.^13,42^

Myopia is related to individual characteristics, but it is also caused by environmental factors and lifestyle, thus prolonged screen use is an important factor in its development.^43^ This should be taken into account and given due importance, since school activities through virtual tools during the pandemic significantly increased screen time, which contributed to symptomatic myopia incidence.^13^

The size of the screen, as pointed out by the studies, can also influence the progression of myopia, devices that have larger screens provide viewing at a more acceptable distance, minimizing the use of close vision, which reduces stimulation of hypermetropic blur, while with smaller screens the distance is shorter, which can lead to increased eye impairment.^44^ These effects are more frequently observed in children and adolescents, since optical development is not fully completed.^13,33^

Based on these findings, it is necessary to disseminate information, especially to parents and guardians, to warn them of the recommendations regarding the necessary distance when looking at a screen, so they can prioritize those that appear to be less harmful and limit use time, since the risks of developing myopia increase every hour that a child is exposed to a digital device.^13^

The change in the lifestyle of the investigated population during the pandemic, with the consequent exacerbated use and the consequent sedentary lifestyle are associated with several health risks, including hypertension, metabolic syndrome and obesity, which presented worrying indicators even before the pandemic.^45^

According to data from the Brazilian Society of Bariatric and Metabolic Surgery, the COVID-19 pandemic has had a major impact on the childhood obesity picture in several countries, including Brazil, due to changing lifestyle habits and increased screen time. The Brazilian Institute of Geography and Statistics (IBGE) points out that, in a group of three children from five to nine years old, one is overweight in the country. The context of obesity in Brazil was already worrisome and worsened during the pandemic. Such reality implies morbidities that can cause various damage to health.^46^

This corroborates the Brazilian study developed in 2022, which sought to investigate the consequences of social isolation in relation to weight gain in children, analyzing the nutritional diagnosis of children in the five Brazilian regions, between the years 2019 and 2021, through reports of the Food and Nutrition Surveillance System (SISVAN) of the Ministry of Health, which observed an increase in weight in children during the pandemic in all Brazilian regions, reflecting changes in lifestyle during this period.^47^ On the other hand, the pandemic showed regional disparities in Brazil, as there were groups of children and adolescents with malnutrition caused by the worsening of the economic crisis, compromising the growth and development of children and youth.^48^

Understanding that these long-term habits can have numerous consequences for this population, it is necessary to develop strategies to reduce screen time and stimulate physical activity by retrieving games that stimulate physical abilities and encouraging sports.

In addition to the increase in sedentary lifestyle, another concern related to the use of screens is poor diet. As well as the implications found in studies for the consumption of foods with low nutritional content and excess calories associated with technologies, other complications arise, which can be justified by the economic crisis faced during this period, the worsening in family income, the increase in food prices, providing food insecurity and malnutrition. Such reality is faced by Brazilian children and adolescents who suffer from neuropsychomotor impairments, that have a greater likelihood of developing chronic diseases such as diabetes, hypertension, and overweight.^49^

The Brazilian Society of Pediatrics points out that during the pandemic families started to overeat foods with high sugar content that provide a sense of pleasure. Despite this, family meals can be a nutritional protection mechanism, by favoring the consumption of complete meals, with appropriate intervals, which can ensure an adequate intake of macronutrients, minerals, antioxidants, and vitamins, thus ensuring adequate nutrition.^50^

Guidelines on food in the school environment or offered by the family members are necessary so that this public is encouraged to create healthier habits in the current transition to the post-pandemic period, an important factor for a good nutritional status, favoring the adequate development of children and adolescents.

Sleep is essential for the proper functioning of the human body, since it contributes to physical and mental homeostasis.^51^ During childhood and adolescence, it gains even more importance by acting in the physiological processes of growth, maturation and health,^52^ in addition to having an important role in strengthening the immune system.^42^

As shown by the investigated studies, the exposure to light-emitting diodes (LED) with a short wavelength (blue range) at night also blocks the secretion of hormones that are important for sleep regulation, such as melatonin, thus morning awakening is later.^53^ In addition, short sleep and early awakening can lead to depressive or stress symptoms.^54^ Thus, it is essential to guide this public about creating routines, having regular and appropriate schedules for sleep, adapting exposure to light, and limiting the use of screens at night.

Thus the importance of stimulating outdoor activities to reduce risks to mental health. Although the quality of mental health is related to numerous factors, controlling exposure to screens is essential.^27^ There is a need to identify the threats to the mental health of this population and to carry out prevention and intervention strategies to minimize the damage caused by confinement as a result of the COVID-19 pandemic.

Among the study's limitations is the fact that analysis of the impacts of screen use during the COVID-19 pandemic was restricted to the month of January 2022, not allowing these impacts to be evaluated up to the present moment. Another limiting factor is the publication of studies in different countries, with only one Brazilian study being retrieved, which shows regional disparities in publications on the subject and makes it impossible to generalize the data. Regarding the levels of evidence, a prevalence of cohort studies, case-control and systematic reviews of qualitative studies was noted. It is believed that the randomized clinical studies not found could clarify the most severe consequences of the use of screens in children and adolescents.

The findings reveal that excessive use of screens during the pandemic has led to numerous consequences for children and adolescents, with a higher incidence of visual impairments, sedentary lifestyle, inadequate eating habits, in addition to weight gain, impaired sleep quality and losses in mental health. The changes in habits due to the restrictive measures presented a contrast between the increase in the consumption of foods with low nutritional content and the excess of calories associated with technology, which points to the problem of overweight and malnutrition that compromises the development of children and adolescents, something that is no longer restricted to developed countries. Thus, this study provides subsidy for health professionals to carry out continuing education focused on this theme, warning parents and guardians of the implications of the use of screens, as well as developing interventions that are effective for children in the prevention and promotion of health in the transition to the post-pandemic period.
